# The Sanatorium Treatment of Tuberculosis

**Published:** 1899-10-21

**Authors:** 


### The Institutional Workshop.

### THE SANATORIUM TREATMENT OF TUBERCULOSIS.

The *West London Medical Journal* for October deals more especially with the British Sanatoria for the Open-air Treatment of Tuberculosis, and gives various details about these establishments. The following are the fees per week which are charged at the various sanatoria mentioned :—

Brinklea Sanatorium, Bournemouth (Dr. Kinsey-Morgan), 3½ to 7 guineas a week.

Cotswold Sanatorium, seven miles from Cheltenham (Dr. Pruen and Mr. C. Braine Hartnell), 5 guineas a week.

Crooksbury Sanatorium, near Farnham, Surrey (Dr. F. Rufenacht Walters), expected to be opened in January, 1900, 5 guineas per week.

"The Firs," Mundesley, Norfolk (Dr. Burton-Fanning), 2½ to 3½ guineas per week.

Linford, Ringwood, Hants (Dr. R. Mander Smyth), 5 guineas per week.

Mount Pleasant, Ventnor, 3 to 3½ guineas per week.

Mundesley Sanatorium, Norfolk (Dr. Burton-Fanning), expected to be ready this month, 5 guineas per week.

Nordrach-upon-Mendip, eight miles from Wells (Dr. Thurnam and Dr. Gwynn), 5 guineas a week.

Overton Hall, Bournemouth (Dr. Francis Pott and Dr. C. Guthrie Stein), 5 guineas a week; the medical fee is extra, 2 guineas a week.

Sunny Mount Sanatorium, Stourfield Park, Bournemouth (Dr. Denton Johns), 4 to 6 guineas per week.

Victoria Hospital for Consumption, Craigleith, Edinburgh (Dr. A. W. Philip).

Woodburn, Morningside, Edinburgh (Dr. W. P. Mears), from 5 guineas per week.

Dr. Jane Walker's Sanatorium, at Boxted, near Colchester, 4 to 5 guineas per week.

The North London Hospital for Consumption.

